# Supplementary material for: Poor Outcomes of Girdlestone Resection Arthroplasty in Injection Drug Users: A Retrospective Study
Source: Antibiotics (Basel). 2024 Aug 21;13(8):782. doi: 10.3390/antibiotics13080782 (PMC11352147; doi:10.3390/antibiotics13080782)
Supplement: Supplementary file 1 [file antibiotics-13-00782-s001.zip › antibiotics-3159338-supplementary.pdf]

Poor Outcomes of Girdlestone Resection Arthroplasty in Injection Drug Users: A Retrospective Study

Henry T. Shu [1,*], Diane Ghanem [1], Oscar Covarrubias [1], Zaid Elsabbagh [1], Alice J. Hughes [2], Rachel B. Sotsky [2], Janet D. Conway [3], Jamie Ferguson [4], Greg M. Osgood [1] and Babar Shafiq [1]

1) Department of Orthopaedic Surgery, Johns Hopkins Hospital, Baltimore, MD 21287, USA
2) Department of Orthopaedic Surgery, Johns Hopkins Bayview Medical Center, Baltimore, MD 21224, USA
3) International Center for Limb Lengthening, Rubin Institute for Advanced Orthopaedics, Sinai Hospital, Baltimore, MD 21215, USA
4) Bone Infection Unit, Nuffield Orthopaedic Centre, Oxford University Hospitals, Oxford OX3 7LD, UK

Henry T. Shu receives postdoctoral funding from the United States National Institute of Arthritis and Musculoskeletal and Skin Diseases.

Babar Shafiq is a paid consultant of Bone Foam Inc and Depuy Synthes. He also receives research support from Depuy. He is a paid speaker and presented for Smith & Nephew. He is on the editorial board of Frontiers in Surgery and is a committee member in the Orthopaedic Trauma Association.

Janet Conway is a paid consultant for Depuy Synthes, Orthofix Inc., and Reselute Medical. She receives research or another financial support from Biocomposites Inc., Bone Support Inc. Depuy Synthes, MHE Coalition, Orthofix Inc., OrthoPediatrics, Pega Medical, Stryker, TRELLIS, and Zimmer. She has stock or stock options with Reselute Medical.

Jamie Ferguson is a paid consultant for DePuy Synthes and a paid speaker for BoneSupport AB, Smith & Nephew.

Greg Osgood is a paid consultant for DePuy Synthes. He is also a board or committee member for the American Academy of Orthopaedic Surgeons and Orthopaedic Trauma Association, an editorial board member for Clinical Orthopaedics and Related Research, Journal of Orthopaedics and Traumatology, Techniques in Orthopaedics, and Wolters Kluwer Health – Lippincott Williams & Wilkins. He also receives research support from Carestream, Siemens, and DePuy Synthes.

The rest of the authors do not have any relevant financial disclosures.

Table of Contents

Page 4. Supplemental Table S1.

Supplemental Table 1. Deidentified descriptions of each patient.

Patient Number	Age	Sex (1 = Male, 2 = Female)	BMI	Received antibiotic spacer	Paraplegic? (1 = Yes, 0 = No)	Active Smoking (1 = Yes, 0 = No)	Indication for girdlestone resection arthroplasty	Blood cultures	Met SIRS Criteria on Presentation? (1 = Yes, 0 = No)	Synovial Aspiration Culture	Local intraoperative antibiotic therapy	Intraoperative tissue cultures	Definitive antibiotic course prescribed by infectious disease	Completed antibiotic course as prescribed by infectious disease	Resolution of hip infection at final follow-up, regardless of previous readmissions	Mortality
1	23	Female	18.39	No	No	Yes	Septic hip with proximal femur and acetabular osteomyelitis without fracture	Negative	No	MRSA	4g vancomycin; 4.8g tobramycin	MRSA	6 weeks of IV Daptomycin	Yes	Yes	No
2	34	Female	17.26	No	No	Yes	Septic hip with proximal femur and acetabular osteomyelitis without fracture	MSSA	No	MSSA	2g vancomycin powder	MSSA	6 weeks of IV oxacillin	Yes	No	No
3	36	Female	25.24	No	No	Yes	Infected hip S/P femoral neck ORIF with hardware failure	Negative	Yes	Negative for growth	1g vancomycin 600mg tobramycin	Negative intraoperative tissue cultures	IV Vancomycin, cefepime, and metronidazole for 6 weeks for broad spectrum coverage due to negative cultures	No	Yes	No
4	38	Male	31.07	No	Yes	Yes	Septic hip with proximal femur and acetabular osteomyelitis without fracture	Negative	No	Negative for growth	Vancomycin and tobramycin, dose not reported	Pantoea septica, candida albicans, VRE	6 weeks with IV daptomycin for E. Faecium, complete prior 6-week course of IV ceftriaxone with Pantoea isolate, metronidazole course and fluconazole course for candida albicans	Yes	No	No
5	40	Male	23.33	No	No	Yes	Infected hip S/P femoral neck ORIF with hardware failure	GAS	Yes	GAS	Vancomycin and tobramycin, dose not reported	GAS, Proteus mirabilis, Arcanobacterium hemolyticum, Enterococcus faecalis Enterococcus faecium, Brevibacterium	6 weeks of IV Zosyn	No	Yes	No
6	43	Male	19.31	No	No	Yes	Septic hip with proximal femur and acetabular osteomyelitis without fracture	MSSA	No	None	1g vancomycin 600mg tobramycin	MSSA	4 weeks of IV Oxacillin	No	No	No
7	44	Female	22.56	No	No	Yes	Septic hip with proximal femur and acetabular osteomyelitis without fracture	Negative	No	MRSA	Vancomycin and tobramycin, dose not reported	MRSA	6 weeks of IV vancomycin	No	No	Yes
8	50	Male	22.5	No	No	Yes	Septic hip with proximal femur and acetabular osteomyelitis without fracture	Negative	Yes	Negative for growth	4g vancomycin; 4.8g tobramycin	Negative intraoperative tissue cultures	6 weeks of IV cefazolin and po Cipro	Yes	Yes	Yes
9	53	Female	18.88	No	Yes	Yes	Septic hip with proximal femur and acetabular osteomyelitis without fracture	Negative	No	No synovial culture done	600mg tobramycin; 1g vancomycin	Negative intraoperative tissue cultures	6 weeks of IV daptomycin, metronidazole, and cefepime	Yes	No	No
10	64	Female	14.69	No	No	Yes	Septic hip with proximal femur and acetabular osteomyelitis without fracture	MSSA	No	MSSA	Vancomycin and gentamicin, dose not reported	MSSA	6 weeks IV cefazolin, later switched to Zo-syn to cover organisms cultured on right forearm wound/osteomyelitis	Yes	Yes	Yes
11	31	Female	22.30	Yes	No	Yes	Septic hip with proximal femur and acetabular osteomyelitis with femoral neck fracture	Negative	No	MRSA	Vancomycin and tobramycin, dose not reported	MRSA	6 weeks of IV vancomycin	Yes	No	No
12	46	Female	28.00	Yes	No	Yes	Infected hip S/P short IMN for hip fracture	Negative	Yes	No synovial culture done	3g vancomycin and 1.2g tobramycin	Negative intraoperative tissue cultures	6 weeks of IV vancomycin and oral ciprofloxacin	Yes	Yes	No
13	47	Male	20.16	Yes	No	Yes	Infected hip S/P femoral neck ORIF with hardware failure	Negative	No	No synovial culture done	3g vancomycin and 1.2g tobramycin	MSSA	6 weeks of IV oxacillin, followed by 3 months of oral cephalexin and rifampin	No	Yes	No
14	50	Female	32.22	Yes	No	Yes	Septic hip with proximal femur and acetabular osteomyelitis without fracture	Negative	No	Micrococcus luteus, cutibacterium acnes	vancomycin, dose not reported	Negative intraoperative tissue cultures	6 weeks of IV vancomycin and cefepime	Yes	Yes	No
15	62	Male	29.97	Yes	No	Yes	Infected hip S/P IMN for hip fracture	Negative	No	No synovial culture done	Vancomycin and tobramycin, dose not reported	Negative intraoperative tissue cultures	6 weeks of IV vancomycin and ertapenem	Yes	Yes	Yes

BMI: Body mass index
S/P: Status post
MSSA: Methicillin-sensitive *Staphylococcus aureus*
MRSA: Methicillin-resistant *Staphylococcus aureus*
GAS: Group A *Streptococci* species
IV: Intravenous
PO: per os
